# Preparation of Extracellular Matrix Protein Fibers for Brillouin Spectroscopy

**DOI:** 10.3791/54648

**Published:** 2016-09-15

**Authors:** Ryan S. Edginton, Sara Mattana, Silvia Caponi, Daniele Fioretto, Ellen Green, C. Peter Winlove, Francesca Palombo

**Affiliations:** ^1^School of Physics and Astronomy, University of Exeter; ^2^Department of Physics and Geology, University of Perugia; ^3^Istituto Officina dei Materiali del CNR, Unità di Perugia

**Keywords:** Bioengineering, Issue 115, Micromechanics, Young's modulus, elasticity tensor, stress, viscoelasticity, hydration, Raman

## Abstract

Brillouin spectroscopy is an emerging technique in the biomedical field. It probes the mechanical properties of a sample through the interaction of visible light with thermally induced acoustic waves or *phonons* propagating at a speed of a few km/sec. Information on the elasticity and structure of the material is obtained in a nondestructive contactless manner, hence opening the way to *in vivo* applications and potential diagnosis of pathology. This work describes the application of Brillouin spectroscopy to the study of biomechanics in elastin and trypsin-digested type I collagen fibers of the extracellular matrix. Fibrous proteins of the extracellular matrix are the building blocks of biological tissues and investigating their mechanical and physical behavior is key to establishing structure-function relationships in normal tissues and the changes which occur in disease. The procedures of sample preparation followed by measurement of Brillouin spectra using a reflective substrate are presented together with details of the optical system and methods of spectral data analysis.

**Figure:**
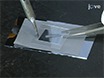


## Introduction

The Brillouin light scattering (BLS) effect was discovered by Léon Brillouin in 1922.^1^ It consists of the inelastic scattering of visible light by thermally activated acoustic phonons in a material. In solid state physics, acoustic phonons are coherent vibrations of all atoms in a lattice. A one-dimensional chain of two alternating types of atoms in a lattice is a simple model illustrating the difference between acoustic phonons, revealed by BLS, and optical phonons, probed by IR absorption and Raman scattering (**Figure 1**). Acoustic phonons are in-phase movements of atoms in the chain with a displacement along the direction of propagation (longitudinal acoustic phonons) or perpendicular to the propagation direction (transverse acoustic phonons), whilst optical phonons are out-of-phase movements of the atoms producing an oscillating electrical dipole moment (longitudinal or transverse modes).

BLS spectroscopy has been used in analytical science since the 1920s; however, only since the 1980s have high contrast measurements been possible through the use of the tandem multipass Fabry-Perot spectrometer. Since then, an increasing number of advances in BLS for analytical applications in condensed matter (where the photon-phonon interaction is exploited)^2-4^ and magnetic materials (through the photon-magnon interaction)^5^ has been brought about. Seminal works on biomedical applications^6-8^ have paved the route to the development of various approaches, including the one applied here and the one described previously^9^ using a reflective substrate in a platelet-like configuration to achieve the full description of the elasticity tensor of a sample.

In the present work, we apply BLS spectroscopy to the fundamental constituents of the extracellular matrix in connective tissues, the fibrous proteins elastin and type I-collagen. Type I collagen is a rigid, triple helical molecule which assembles laterally and longitudinally with extensive cross linking to form essentially rigid fibers in tissues such as tendons. Networks of collagen often co-exist with networks of elastin, a protein which, unusually, generates long range elasticity through a combination of entropy and hydrophobic interactions with its environment and is essential to the functions of tissues such as lung and skin. Both fibers are modeled using a hexagonal crystal model in the current research.^9^ In part 1, we describe the protocol to extract the fibers from animal tissues and to prepare the sample for the spectroscopic measurements. In part 2, the procedure for setting up the Brillouin apparatus and acquiring spectra from the fibers is presented. Part 3 gives details of data analysis applied to the Brillouin spectra to extract the relevant mechanical information contained therein. Then, representative results are presented and discussed.

## Protocol

Caution: Please consult biological safety protocols and all relevant material safety data sheets (MSDS) before use. The laser employed in these experiments is a Class 3B laser; compliance with local rules for a safe use of the system is required. Please use all appropriate safety practices when performing a laser spectroscopy measurement including the use of personal protective equipment (safety goggles).

Tail tendons were obtained from 7-8 week old Wistar rats euthanized for other purposes in accordance with EU regulation 1099/2009 and The Welfare of Animals (slaughter or killing) Regulations 1995. Bovine nuchal ligaments were obtained from a local abattoir.

### 1. Preparation of Sample Fibers

NOTE: Protein fibers of the extracellular matrix can be extracted from various tissues, using different procedures. Protocols were refined based on widely applied procedures.

1. **Extraction of collagen fibers from rat tail**
  1. Sacrifice a rat by intra-peritoneal injection of 100 mg/kg of body weight sodium pentobarbitone. Then sever the tail directly at the point of contact with the body, pressing down with a single-edge razor blade. Wrap the tail in cling film and store it frozen at -20 °C until required.
  2. Collect the tail from the freezer, cut a 20 mm long segment from the proximal end whilst still frozen and then leave it to thaw in a Petri dish filled with phosphate buffered saline (PBS) solution (pH 7.4) at RT.
  3. Once the tail is thawed, make an incision along the length of the segment using a scalpel to split the skin. Then peel back to reveal four sheathed tendon bundles about the tail vertebra.
  4. Using fine forceps and being careful not to apply any pre-strain, gently draw each fiber out of the sheath and place it in a vial containing distilled water with 0.01% w/v sodium azide (NaN_3_) to prevent bacterial growth, and store refrigerated. A single tail yields around thirty tendon fibers.
  5. To obtain pure fibrous type I collagen, apply a three part enzymatic digestion process^10^ to the tendon fibers to remove the proteoglycans and all other noncollagenous material.
    1. First, immerse the fibers in 0.125 U/ml chondroitinase ABC in 0.05 M Tris buffer and 0.06 M sodium acetate (CH_3_COONa) at pH 8.0 for 24 hr at 37 °C in a shaking incubator at 200 rpm.
    2. Then, immerse the fibers in 1 U/ml streptomyces hyaluronidase in 0.05 M Tris buffer and 0.15 M sodium chloride (NaCl) at pH 6.0 and return to the shaking incubator for 24 hr at 37 °C.
    3. Finally, immerse the fibers in 1 mg/ml of trypsin in 0.05 M sodium phosphate (NaHPO_4_) and 0.15 M NaCl at pH 7.2 for 16 hr in the shaking incubator at 37 °C.
  6. Store the purified fibers refrigerated in vials containing distilled water, with 0.01% NaN_3_ to prevent bacterial growth, until required for measurement.
2. **Extraction of elastin fibers from bovine nuchal ligament**
  1. Obtain bovine nuchal ligament from the abattoir, wrap it in cling film and store it frozen at -20 °C until use.
  2. To produce pure elastin, collect the ligament from the freezer, allow it to thaw at RT, defat it using a scalpel and digest the ligament in a boiling water bath according to the Lansing procedure,^11^ as follows.
    1. Prepare a 0.1 M solution of sodium hydroxide (NaOH) in distilled water and add it to the defatted ligament in a conical flask, covering the tissue.
    2. Boil the flask in a water bath at 95 °C for 45 min.
    3. Remove the ligament from the digestion flask and wash the insoluble tissue block repeatedly in distilled water until pH 7.0 (monitored using a pH meter) is obtained.
    4. Remove the tissue from the final wash solution and submerge it in distilled water (mixed with 0.01% NaN_3_ to prevent bacterial growth) in a sealed container and store it refrigerated.
  3. Collect the tissue from the refrigerator and, being careful not to apply too much force and pre-strain, use tweezers to gently pull smaller elastin segments (20 to 50 mm long, *ca.* 2 mm thick) away from the larger block and place them in a Petri dish with PBS solution (pH 7.4).
  4. Using fine forceps, gently tease small fiber bundles around 1 mm thick and cut them to lengths of a few mm using a scalpel.
  5. Transfer the fibers to vials containing distilled water (with 0.01% NaN_3_ to prevent bacterial growth) and store them refrigerated until required for measurement.
3. **Mounting the fibers onto reflective substrate**
  1. Using a diamond cutter, cut a piece of reflective silicone slide.
  2. To create a hydrated compartment, cut a strip of Parafilm to fit over the silicone slide with a hollow cut in the center (large enough to fit a fiber) and place it onto the silicone substrate. NOTE: For dry fiber measurements, cut the parafilm into a U-shape so that one of the four sides stays open to the air when sealed in step 1.3.4.
  3. Remove the fibers from the refrigerator, use a pair of fine forceps to collect a single fiber from the storage solution and place it in a small Petri dish filled with pure water at RT for 5 min to wash the sample. Then, collect the fiber and transfer it to the center of the parafilm hollow on the silicone substrate. Careful: Avoid damaging the fiber by stretching it during this operation, and avoid reorienting the sample on the substrate as this may cause a change in mechanical properties.
  4. Place a thin glass coverslip over the fiber and seal the chamber by passing a heated soldering iron gently over the glass surface to melt the parafilm beneath the glass. Careful: Avoid damaging the fiber by not bringing the soldering tip too close or heating the substrate excessively.
  5. Place the sealed chamber on a flat surface under a small weight and leave it for around 12 hr to achieve a good contact between the sample and silicon substrate while avoiding damage to the specimen.
  6. Remove the weight and secure the chamber in place on the substrate, using screws.

### 2. Setting up the Brillouin Experiment and Acquiring Fiber Spectra

1. **Preparation of sample compartment**
  1. Mount the sample prepared as in part 1.3 onto a vertical holder equipped with a goniometer to enable in-plane rotation of the sample while maintaining a constant scattering angle (2*Φ* = 90°; see **Figure SI-1**) and scattering volume position.
  2. Perform an accurate focus adjustment of the laser light on the sample through the lens.^9^ Careful: The laser output power may be too high and produce a burn in the sample. Make sure that it is set sufficiently high to give a good sensitivity but not too high to avoid damage to the sample. Here we used a power of *ca.* 76 mW on the sample. This was sufficient to get a good sensitivity without burning the sample, also considering that this is thin and in contact with a substrate that helps dissipating the heat generated by laser illumination.
  3. Position the sample at a 45° angle (*Φ*) to the incident laser beam using a Vernier scale. Achieve optimal positioning by running a measurement and maximizing the intensity of the peaks in the spectrum (see below).
2. **Setting up the spectrometer**
  1. Open the software for acquisition and manipulation of the data and set up the acquisition of a Brillouin spectrum of the sample^12^. The procedure described here applies to the multipass tandem interferometer (**Figure SI-1A**).
  2. Align the two Fabry-Perot (FP) interferometers independently changing the voltages applied to the piezo by the control unit. For this pre-alignment procedure, observe the light reflected by each FP. When the intensity reflected by the two FPs tends to zero, the correct alignment is reached.
  3. Calibrate the spectrum: the accessible frequency range, or *free spectral range* (FSR), is dependent on the distance between the two mirrors of the first FP cavity, *L*, through FSR = *c*/2*L*, where *c* is the speed of light and *L* is measured by a dial-gauge.
  4. Synchronize the scans of the two FP interferometers and switch the optical system to the tandem multipass configuration. A feedback control of the transmitted laser light intensity will automatically maintain the alignment of the two FPs during the measurement.
3. **Measurement of Brillouin Spectra** Careful: The Brillouin spectrum is highly dependent on the temperature and hydration of the sample and so careful control of these parameters is key to obtaining reproducible spectra.
  1. Start the acquisition of a Brillouin spectrum of the sample and run it until a good signal-to-noise ratio is achieved. This may take several minutes depending on the scattering cross section, concentration and thickness of the sample. NOTE: There is not a rule of thumb for the signal-to-noise ratio but the spectral quality is checked by the experimentalist based on the specific sample analyzed. There is a trade-off between spectral quality and duration of the measurement, therefore the experimental parameters need to be selected according to the specific application.
  2. For the measurement of a dry specimen, take successive spectra — for each of them, following step 2.3.1 — until no changes in the position of the peaks is observed. This is achieved when the sample is at equilibrium with the room atmosphere and no further drying will affect the spectrum.
  3. Select the light polarization (VV or VH; V stands for vertical and H for horizontal direction of the light polarization relative to the scattering plane) and acquire spectra at each angle to fiber axis (*θ *; **Figure SI-1**) by rotating the sample in plane by hand.
  4. Save the Brillouin spectra to file for subsequent processing.

### 3. Analysis of Brillouin Spectra

NOTE: Fit analysis of Brillouin peaks can be performed using various functions. A damped harmonic oscillator (DHO) function^4,13^ was selected as this is a valid model for peaks originating from damped acoustic modes in viscoelastic media.

1. **Fit analysis of Brillouin peaks**
  1. Select the spectral range for the peak of interest in the Brillouin spectrum.
  2. Enable a baseline in the fit if the spectral background is much higher than zero. NOTE: The baseline can vary between spectra. Ensure that the correction is applied in a systematic and reproducible manner.
  3. Apply a detailed least squares fitting using a DHO function^4,13^ to the Brillouin peak of interest iteratively until convergence is achieved and the best fitting curve is obtained. Then, save the fit results to file.
  4. Obtain average values from the fit parameters of the two peaks of each Brillouin doublet.
  5. Calculate the acoustic wave velocity from the peak frequency (using the expression below).
  6. Plot the fit results through graphs, *e.g., *acoustic wave velocity vs. angle to fiber axis, *θ*, and apply relevant models (*e.g., *for acoustically anisotropic systems^9^) to extract mechanical quantities such as elasticity tensor coefficients.

## Representative Results

The Brillouin spectroscopy apparatus used in this experiment (**Figure SI-1A**) has been previously described.^9^ It employs a single-mode 532 nm solid-state laser with 76 mW output power at the sample. A 20 cm achromatic lens focuses the laser light onto the sample and collects the scattered light from the sample in a backscattering geometry. A tandem multipass Fabry-Perot interferometer is used for filtering the scattered light, which is then detected by a low-noise photodiode detector. This approach gives extremely high contrast (*ca.* 120 dB) and stability through self-aligning piezo-scanning of the etalons. A polarizer and analyzer are introduced to select the polarization of the incident and scattered light. Spectra are usually obtained with the polarizer kept fixed selecting the vertical (V) direction of the incident light polarization and the analyzer selecting alternatively the vertical (V) or horizontal (H) direction of the scattered light polarization. In this configuration, longitudinal and transverse acoustic modes are detected respectively.

A typical Brillouin spectrum has an intense central peak due to elastic scattering and one or more sets of equally shifted peaks, or *Brillouin doublets*, which are the signature of the mechanics of the sample. In these measurements, the scattered light can originate both from bulk phonons travelling quasi-orthogonal to the sample and, after reflection of the incident light at the sample-substrate interface, from bulk phonons travelling parallel to the surface (PS modes).^9^

**Figure 2** shows BLS spectra of dry and hydrated trypsin-digested collagen fibers obtained with VV polarization at 0.2 GHz resolution, with a 30 GHz free spectral range and approximately 10 min collection time per spectrum. Each spectrum corresponds to a specific angle of rotation, *θ*(**Figure SI-1C**). In dry collagen fiber at *θ * = 0°, longitudinal modes give rise to a bulk peak at (18.92 ± 0.02) GHz whilst the PS mode is at (9.85 ± 0.03) GHz (**Figure 2A**). The PS peak shifts to lower frequencies as *θ * goes from 0° (phonon probing the axial orientation of the fiber) to 90° (phonon probing the radial orientation), whereas the bulk peak only slightly red-shifts upon changing *θ * in the same range (phonon probing a quasi-radial direction throughout the rotation). In wet collagen fiber, the two peaks due to longitudinal phonons are essentially unchanged throughout the experiment, with the bulk peak at *ca.* 10.5 GHz and the PS peak at 4.9 GHz (**Figure 2B**). This indicates an 80 to 100% reduction in peak frequency (relative to the data of 18.92 and 9.85 GHz, respectively), and hence of stiffness of the material, due to hydration. Note that the bulk and PS modes of hydrated collagen lie close in frequency to the modes of pure water, suggesting that its elastic constants are a combination of the water and fiber contributions, with a dominant role played by water.

**Figure 3** shows a spectrum of dry trypsin-digested collagen fiber measured at *θ * = 30° with VH polarization; a leakage of the VV polarization enables the PS and bulk peaks to still be observed. Transverse modes account for a peak at (4.1 ± 0.2) GHz (*θ * = 0°) which slightly blue-shifts as *θ * changes from 0° to 90°. Fit results for both the transverse and PS peaks are also shown. Peak parameters were extracted and acoustic wave velocities were derived as *V*_L_ = *v*λ/√2, where *v* is the frequency of the mode obtained by curve-fit analysis of the peaks and λ is the excitation wavelength, 532 nm. Note that in this geometry, knowledge of the refractive index of the material is not required to obtain the acoustic mode velocity (owing to the scattering geometry, *q_s_* = 2*k_i_* sin(*Φ*); **Figure SI-1b,c**), hence making this approach especially advantageous.

**Figure 4** is a plot of the acoustic wave velocities obtained from longitudinal and transverse modes (PS and T peaks) as a function of the angle θ**. Fit analysis to a model of hexagonally symmetric elastic solid^7^ — Equations A1 and A2 below — provides the five components of the elasticity tensor of dry trypsin-digested type I collagen fibers (**Table 1**).

The longitudinal and transverse acoustic wave velocities are given by^9^


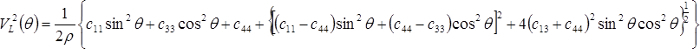
, (A1)


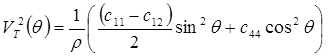
, (A2)

where ρ is the density of the material, and *c*_11_, *c*_33_, *c*_44_ and *c*_13_ are four of the five elastic constants that characterize systems with a hexagonal symmetry. The fifth constant, *c*_12_, can be derived from the approximate relation *c*_12_ ~ *c*_11_ - 2*c*_44_.^7^

Coefficients are similar to those previously obtained from unpurified collagen fibers.^9^ A noticeable difference occurs for the coefficient *c*_13_ that is reflected into similar values of the elastic moduli *E*_ǁ_ and *E*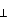 (approximately 7.2 and 7.7 GPa) for the purified collagen.

**Figure 5** is a plot of the longitudinal acoustic wave velocity of wet collagen versus *θ*. In this case, no periodic change in frequency is observed, giving a constant velocity within the error. **Figure 6** shows the spectra of dry and hydrated elastin fibers measured at *θ * = 0°. Transverse modes were not detected for these samples. In dry elastin, the bulk peak occurs at 16.8 GHz whilst the PS mode at 8.2 GHz^9^ (13 and 20% lower than the corresponding peaks of dry collagen). Wet elastin fibers present a bulk peak at (12.30 ± 0.01) GHz (37% lower in frequency than the bulk peak of dry elastin). The PS mode of wet elastin is not apparent in the spectrum because of the intense tail of the elastic peak at those frequencies. On the other hand, the peak at *ca.* 7.5 GHz is attributed to the bulk water.

**Figure 7** shows the dependence of acoustic wave velocity in dry elastin fiber on *θ*. From these data, the elasticity tensor components (and mechanical moduli) were obtained (**Table 1**).^9^ As in wet collagen, there is evidence of isotropy in the mechanical modulus of hydrated elastin fibers. These results indicate how Brillouin spectroscopy can give relevant information on stiffness, composition and structural aspects of a material.


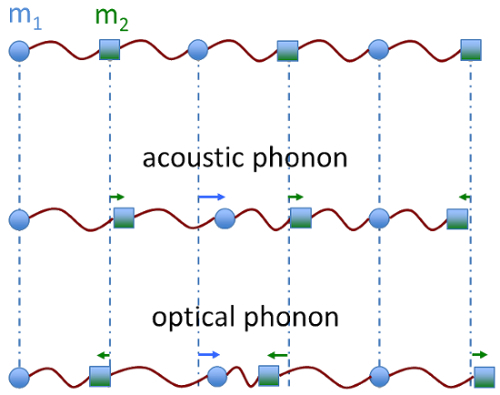
**Figure 1.Acoustic and optical phonons in one-dimensional chain of atoms.** Schematic diagram of acoustic and optical vibrations in a one-dimensional diatomic chain. Atoms have mass m_1_ and m_2_ and are alternated. Arrows indicate the displacements of the atoms. Please click here to view a larger version of this figure.


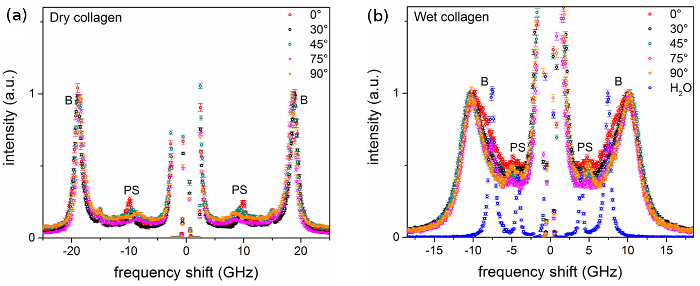
**Figure 2.Brillouin spectra of trypsin-purified type I collagen fibers from rat tail tendon.** Spectra of (**A**) dry fiber and (**B**) hydrated fiber from VV measurements at different angle to fiber axis *θ*, in degrees. A spectrum of pure distilled water is also shown. Spectra were normalized to the intensity (height) of the bulk peak. Labels B and PS denote peaks related to bulk and parallel-to-surface modes, respectively. Error bars indicate the standard error (square root of number of counts). Please click here to view a larger version of this figure.


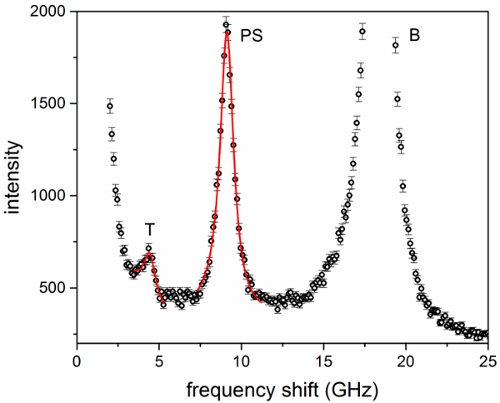
**Figure 3.Brillouin spectrum of dry trypsin-purified type I collagen fibers from rat tail tendon.** Spectrum from a VH measurement at *θ*= 30°. Labels T, PS and B denote peaks related to transverse, parallel-to-surface and bulk modes, respectively. Results of fit analysis using a damped harmonic oscillator (DHO) model for both T and PS modes are also shown. Error bars indicate the standard error (square root of number of counts). Please click here to view a larger version of this figure.


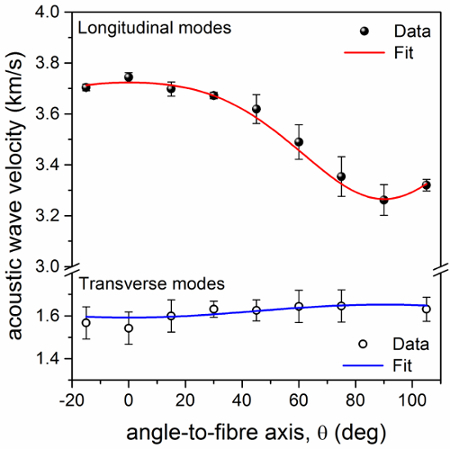
**Figure 4.Plot of the acoustic wave velocity in dry trypsin-purified collagen vs angle to fiber axis.** Longitudinal and transverse acoustic wave velocities of dry collagen fiber derived from fit analysis of the Brillouin peaks. Data are fitted to a model of hexagonally symmetric elastic solid. Red line: Equation A1 (*R*^2^ = 0.99); blue line: Equation A2 (*R*^2^ = 0.36). Error bars indicate the standard errors obtained from the square root of the diagonal elements of the covariance matrix after a Levenberg-Marquardt nonlinear least squares fit of Brillouin spectra. Please click here to view a larger version of this figure.


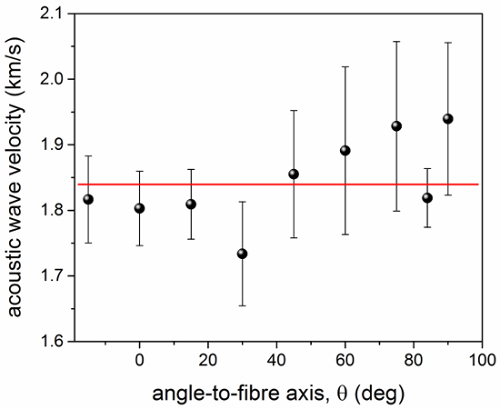
**Figure 5.Plot of the longitudinal acoustic wave velocity in wet trypsin-purified collagen vs angle to fiber axis.** Longitudinal acoustic wave velocity of hydrated collagen fiber derived from fit analysis of the Brillouin peaks. The line shown is a guide for the eye and gives the average value of the acoustic wave velocity in this range. Error bars indicate the standard errors obtained from the square root of the diagonal elements of the covariance matrix after a Levenberg-Marquardt nonlinear least squares fit of Brillouin spectra. Please click here to view a larger version of this figure.


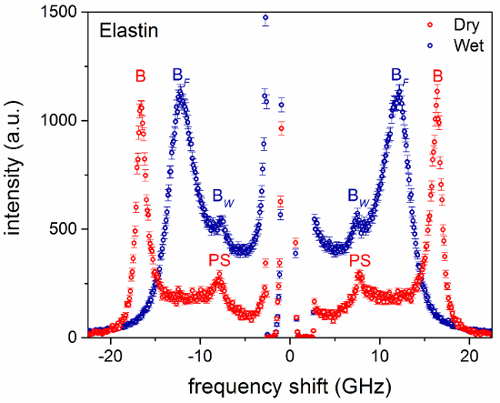
**Figure 6.Brillouin spectra of elastin fibers from bovine nuchal ligament.** Spectra of dry and hydrated fiber at *θ* = 0°. Spectra were normalized to the intensity (height) of the bulk peak. Labels B and PS denote peaks related to bulk and parallel-to-surface modes, respectively. B*_F_* and B*_W_* refer to the bulk peaks of the fiber and water, respectively. Error bars indicate the standard error (square root of number of counts). Please click here to view a larger version of this figure.


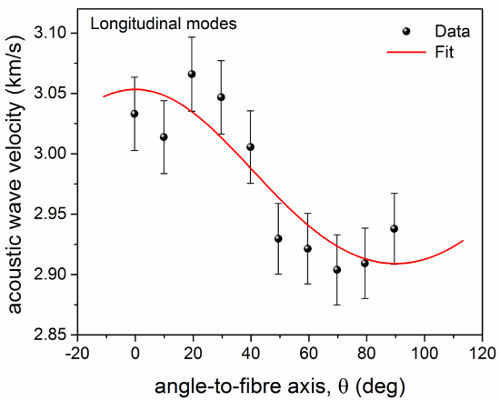
**Figure 7. Plot of the longitudinal acoustic wave velocity in dry elastin vs angle to fiber axis. **Longitudinal acoustic wave velocity of dry elastin fiber derived from fit analysis of the Brillouin peaks. Data are fitted to a model of hexagonally symmetric elastic solid. Red line: Equation A1^9^ (*R*^2^ = 0.74). Error bars indicate the standard errors obtained from the square root of the diagonal elements of the covariance matrix after a Levenberg-Marquardt nonlinear least squares fit of Brillouin spectra. Please click here to view a larger version of this figure.


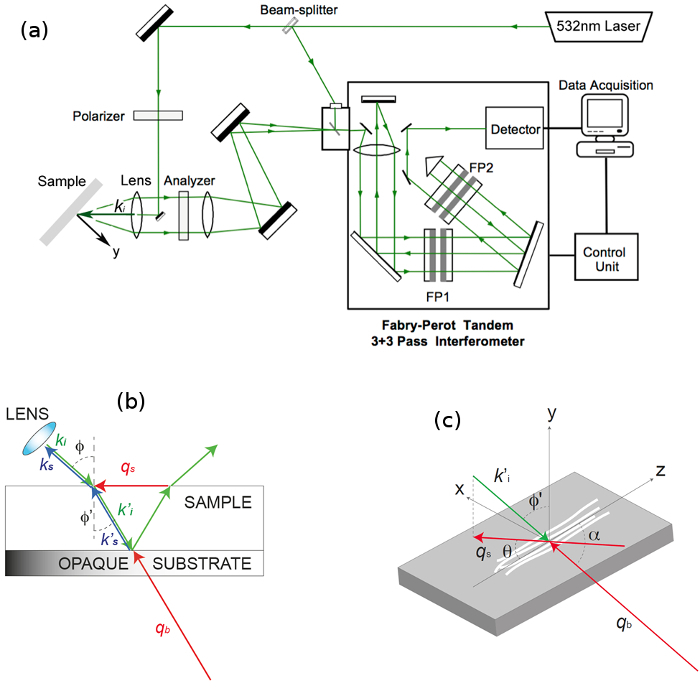


**Figure SI-1. Schematic of the Brillouin set-up and BLS scattering geometry.** (**A**) The incident light emitted by a solid-state laser is sent to the sample through an achromatic lens. The light scattered by bulk acoustic phonons and by those resulting from reflection of light at the substrate surface, which is in contact with the sample, is collected by the lens, filtered by a tandem-multipass Fabry-Perot interferometer and detected by a photomultiplier. FP1 and FP2 indicate the two interferometers constituting the tandem set-up. A polarizer selects the polarization of incident light, and an analyzer is used to select the polarization of scattered light. (**B**) BLS geometry with a specimen in contact with the surface of a reflective silicon substrate. A glass slide (not shown) is placed above the specimen to seal the compartment, and gentle pressure is applied through pads at the corners of the substrate. The incident light (*k*_i_) passes through the lens, is refracted at the air-sample interface (*k'*_i_) and focused at the sample-substrate interface. The scattered light collected by the same lens (*k'*_s_) results from interaction with both bulk phonons (*q*_b_) and those travelling PS of the sample (*q*_s_). Angles between the directions of light and the normal to the surface are indicated as *Φ* and *Φ'. *(**C**) Schematic diagram of the sample and of the adopted coordinate system*; z* defines the extraordinary axis parallel to the direction of the fibres. Angles *θ* and *α* are those between the direction of phonons *q*_s_ and *q*_b_ to the *z*-axis, respectively. *k*_i_, *k'*_i_, *k*_s_, *k'*_s_: wavenumbers of the incident and scattered light; *q*_b_, *q*_s_, wave vectors of the bulk and PS modes, respectively. (Reprinted from ref 9.) Please click here to view a larger version of this figure.

**Table 1. Elastic tensor coefficients derived from fit analysis of the acoustic wave velocities.** Elastic tensor coefficients of dry trypsin-purified type I collagen fibers (this work) and elastin fibers (ref 9).

**Table:** 

**sample**	**elastic coefficients (GPa)**
trypsin-digested collagen	c_33_	18.7 ± 0.1
	c_11_	14.4 ± 0.2
	c_44_	3.4 ± 0.1
	c_12_	7.2 ± 0.2
	c_13_	11.2 ± 0.3
elastin	c_33_	11.5 ± 0.2
	c_11_	10.4 ± 0.1
	c_44_	1.9 ± 0.2
	c_12_	6.6 ± 0.2
	c_13_	6.8 ± 0.3

## Discussion

Brillouin scattering spectroscopy is a unique tool by which the individual components of the elasticity tensor of a protein fiber can be characterized in unprecedented detail. Furthermore, the measurements can be made on a microscopic scale and thereby will provide us with novel insights into the micro-scale mechanics of biological structures, allowing us, for the first time, to understand the mechanical, and probably functional, significance of the complexities in matrix architecture and biochemistry which has been revealed in recent years.

The technique measures mechanical properties in a GHz frequency range. This domain has never been explored before for structural biopolymers and it both raises and provides the means to answer fundamental questions about molecular mechanisms of elasticity.

We described the steps to extract collagen and elastin fibers from animal tissues and to measure Brillouin scattering spectra using a reflective substrate to achieve the complete description of fiber biomechanics. Critical steps within the protocol are those that ensure that purified fibers are obtained and appropriate experimental conditions are in place for reproducible measurements of the fibrous proteins. However, it must be borne in mind that the extraction procedures may modify the mechanical properties of the fibers.

Modifications of the technique involve the coupling with optical microscopy for microfocused Brillouin scattering and mapping approaches^13^ and the possible combination with complementary techniques (*e.g.,* Raman scattering). Current applications of the technique are mainly focused on excised biological materials, but important developments, *e.g.,* those based on multiple VIPA etalons^14^, are making possible the translation of this technique from the benchtop to the bedside with a range of applications already demonstrated^15,16^ including potential *in vivo* applications. The VIPA approach is an alternative to what we describe; it has faster acquisition time but is not necessarily appropriate in the case of opaque samples such as those analyzed here. Moreover, the use of a reflective substrate is not practical in set-ups that use the VIPA etalons because their contrast would not be sufficient to reject the quasi-elastic light. Limitations related to the speed of acquisition of a spectral dataset and the inherently weak scattering cross section of the material may limit applications to dynamic biological systems and to the acquisition of data from deep within tissues, but technical refinements may improve on current performance.

BLS promises to be a major tool in fundamental biophysical research on the extracellular matrix and thereby to produce new insights into the evolution of mechanical properties during matrix growth and their loss in pathological degeneration. However, it is important to remember that the measurements are noninvasive and might therefore be undertaken *in vivo*. Indeed, this has already been achieved in the cornea^16^ and such work may provide a platform for the development of new diagnostic tools for a wide range of connective tissue disorders.

Ultrasound elastography and atomic force microscopy (AFM) are alternative methods of micromechanical measurement, but the BLS technique offers better spatial resolution (on a subcellular scale) than the former and, unlike AFM, imposes no mechanical forces on the specimen and is not restricted to the analysis only of surface features. Brillouin moduli of collagen and elastin are in the GPa range, whilst Young's moduli from macroscopic strains are of the order of MPa (further details will be reported elsewhere). This result indicates a differential elastic modulus with a strong dependence on the excitation frequency, owing to the viscoelastic behavior of the fibers. BLS can be applied to a wide range of problems and materials in biomedical science. It can help in answering questions on physiology and pathology of biological tissues, as well as provide a physical tool for the fundamental understanding of materials and interactions at the molecular level.

## Disclosures

The authors have nothing to disclose.
